# 

**Published:** 1868-07

**Authors:** 


*The Printing of the Journal.*—Through the enterprise of the publishers, the *American Journal of Dental Science* is presented to our readers in new type, and materially improved in appearance.

This improvement is due to the energy of the publishers, who have established a *printing office* in their Dental Depot, with a view ot getting up the *Journal* in the best manner possible; and also to furnish the patrons of their Depot with professional cards, appointment lists, dental account books, &c. This number of the *Journal* is a success so far as mechanical execution is concerned, and a specimen of what the office is able to perform. We are assured that our readers will agree with us in saying that Messrs. Snowden & Cowman have good cause to congratulate themselves on the success which has thus far attended their efforts to enhance the *Journal*, by establishing an office for its publication.

---

*Time of Meeting of American Dental Association.*—The eighth session of this Association will be held in Grant's Hall, Niagara Falls, beginning on Tuesday, July 28th 1868.

The following arrangements have been made with regard to accommodations. The International Hotel will receive members of the Association at $4.00 per day, a reduction of fifty cents per day from their regular terms. The Spencer House charges $3.50 per day. By giving timely notice to the committee of arrangements, apartments will be reserved for members of the Association, especially those accompanied by their families, at either of these hotels.

Geo. B. Snow,<br>B. T. Whitney,<br>A. P. Southwick, } Committee of Arrangements.

Buffalo, N. Y.

